# High-Definition Intravascular Ultrasound Versus Optical Coherence Tomography: Lumen Size and Plaque Morphology

**DOI:** 10.1016/j.jscai.2024.102520

**Published:** 2025-05-01

**Authors:** Wei Wu, Shijia Zhao, Akshat Banga, Yash Vardhan Trivedi, Vineeth S. Dasari, Parth Munjal, Rakshita Ramesh Bhat, Ruben K.A. Tapia-Orihuela, Usama M. Oguz, Hammad Zafar, Haritha Darapaneni, Nikolaos Spilias, Jessica Wagner, Stephen Morin, Amanda DeVos, Paul A. Iaizzo, Akiko Maehara, Evan S. Shlofmitz, Ziad A. Ali, Emmanouil Brilakis, George D. Dangas, Thomas Johnson, Yiannis S. Chatzizisis

**Affiliations:** a Center for Digital Cardiovascular Innovations, Cardiovascular Division, Miller School of Medicine, Miami, Florida; b Department of Chemistry, University of Nebraska–Lincoln, Hamilton Hall, Lincoln, Nebraska; c Visible Heart Laboratories, Department of Surgery and the Institute for Engineering in Medicine, University of Minnesota, Minneapolis, Minnesota; d Division of Cardiovascular Medicine, Columbia University Irving Medical Center, New York, New York; e Division of Cardiovascular Medicine, St. Francis Hospital & Heart Center, Roslyn, New York; f Center for Complex Coronary Interventions, Minneapolis Heart Institute, Minneapolis, Minnesota; g Division of Cardiovascular Medicine, Icahn School of Medicine at Mount Sinai, New York, New York; h Bristol Heart Institute, Translational Health Sciences, University of Bristol, Bristol, United Kingdom

**Keywords:** coronary artery, high-definition intravascular ultrasound, microcomputed tomography, optical coherence tomography

## Abstract

**Background:**

Complex percutaneous coronary interventions rely on advanced intravascular imaging techniques, such as intravascular ultrasound (IVUS) and optical coherence tomography (OCT). This study aims to compare the accuracy of the latest high-definition intravascular ultrasound (HD IVUS) and OCT technologies in measuring coronary lumen dimensions and assessing plaque morphology.

**Methods:**

We compared HD IVUS and OCT in 8 silicone models using microcomputed tomography as the ground truth. We also compared HD IVUS vs OCT in 12 coronary arteries from 9 patients.

**Results:**

In the silicone models, the latest HD IVUS (AVVIGO+, Boston Scientific) system overestimated lumen dimensions compared to microcomputed tomography by +0.06 ± 0.05 mm for mean lumen diameter (MLD). OCT (Ultreon 2.0, Abbott) underestimated lumen dimensions by –0.17 ± 0.06 mm for MLD, with the underestimation being greater for lumen diameters ≥5.0 mm. In clinical cases, the latest HD IVUS (AVVIGO+) system yielded larger lumen dimensions compared to OCT (Ultreon 2.0) by +0.12 ± 0.11 mm for MLD, and the earlier HD IVUS (POLARIS, Boston Scientific) system also showed larger lumen dimensions compared to OCT (AptiVue, Abbott) by +0.26 ± 0.29 mm for MLD. Using OCT as the reference, HD IVUS detected fine wall structures with precision, such as a thin fibrous cap, dissection, and stent struts.

**Conclusions:**

The latest HD IVUS (Avvigo+) tends to overestimate lumen size, whereas OCT (Ultreon 2.0) underestimates it. Experimental data suggest HD IVUS provides more accurate lumen assessment, particularly in larger coronary arteries, although both technologies exhibit comparable overall accuracy in the clinical setting.

## Introduction

The efficacy of complex percutaneous coronary interventions depends significantly on obtaining precise intravascular imaging to make informed clinical decisions and improve overall procedural outcomes.^1^ Studies have demonstrated that image-guided percutaneous coronary intervention is superior to traditional angiography in enhancing the accuracy and effectiveness of coronary interventions.^2^ Imaging modalities, such as intravascular ultrasound (IVUS) and optical coherence tomography (OCT) provide detailed, accurate imaging of the coronary arteries, surpassing the performance of coronary angiography.^3^ High-definition intravascular ultrasound (HD IVUS) employs 60 MHz ultrasound technology that delivers high spatial resolution (20-40 μm) making it particularly effective for evaluating larger coronary arteries and deeper vessel layers. OCT, utilizing near-infrared light, offers superior spatial resolution (10-20 μm), which is essential for visualizing the fine structures of the vessel wall and plaque composition.^4^

Several studies have compared IVUS and OCT in measuring coronary lumen dimensions.^4^, ^5^, ^6^, ^7^, ^8^, ^9^, ^10^, ^11^, ^12^, ^13^, ^14^ Only 3 studies (2 in vitro and 1 clinical) have specifically examined HD IVUS technology vs OCT. These studies used earlier technologies of HD IVUS (OPTICROSS HD/iLab, Boston Scientific; Kodama, ACIST) and OCT (Dragonfly OPTIS/AptiVue, Abbott).^6^^,^^9^^,^^10^ However, the accuracy of the latest and most widely used HD IVUS (OPTICROSS HD/AVVIGO+, Boston Scientific) and OCT (Dragonfly OPTIS/OPTIS/Ultreon 2.0, Abbott) technologies have not been studied. In the current study, we performed a comprehensive comparison of the latest HD IVUS with OCT in the following: (1) silicone models using microcomputed tomography (μCT) as ground truth, and (2) clinical cases. We focused on lumen size and plaque morphology as comparison metrics.

## Materials and methods

All methods were performed following the relevant guidelines and regulations. The study was approved by the University of Nebraska Medical Center Institutional Review Board (approval number 0794-17-FB), and written informed consent was obtained from all subjects.

### In vitro experimental analysis

#### Silicone models

Using a previously described method,^15^ we constructed 8 cylindrical silicone models with diameters as follows: 2.0 mm, 2.5 mm, 3.0 mm, 3.5 mm, 4.0 mm, 4.5 mm, 5.0 mm, and 6.0 mm. The length of the models was 16.5 ± 1.0 mm. For each model, we designed a negative mold using 3D computer-aided design software Rhinoceros 6 (Robert McNeel & Associates), and these molds were 3D printed in acrylonitrile butadiene styrene using a Stratasys Dimension Elite 3D printer (Stratasys) at a resolution of 178 μm. The inner surface of the molds was smoothed with acetone vapor. Polydimethylsiloxane was then poured into the molds along with a curing agent and cured for 48 hours at 65 °C, after removing air bubbles under vacuum. Acrylonitrile butadiene styrene was subsequently dissolved in acetone using an ultrasonic cleaner (Branson 1800, Cleanosonic), leaving them ready for use.

The silicone models were then integrated into a custom-built flow chamber, with connections established at both the inlet and outlet. Each model was subjected to a continuous flow of blood-mimicking fluid (Doppler test fluid, CIRS) for HD IVUS scanning, or distilled water for OCT scanning. The flow was maintained at a constant rate of 100 mL/min and conducted at room temperature.

#### μCT imaging

To establish a ground truth for the quantitative comparison of HD IVUS and OCT, the silicone models were scanned using μCT (North Star Imaging, X3000). The scanning parameters were an image pixel size of 23.76 μm, voltage of 132 kV, current of 180 μA, and slice thickness of 27 μm. Iodinated contrast media was utilized within the model lumen to enhance the visualization of the lumen border under X-ray imaging. The silicone models were subsequently reconstructed in 3D from the μCT images using a 3D medical imaging software Materialise Mimics 24.0 (Materialise). The 3D reconstructed models were then sectioned into equidistant segments (0.5 mm intervals) and quantitatively analyzed using Rhinoceros 7.0.

#### HD IVUS image acquisition and analysis

The silicone models were imaged using HD IVUS with a 60 MHz HD OPTICROSS 6 catheter and AVVIGO+ system. Technical specifications and device parameters for HD IVUS are summarized in Supplemental Table S1. For each model, the HD IVUS catheter was advanced beyond the distal edge of the model under direct angiographic visualization and then pulled back at a constant speed of 1.0 mm/s. For HD IVUS, coregistration was achieved by identifying corresponding landmarks on HD IVUS images. This method provided a consistent and reliable basis for coregistration, allowing for valid comparisons of measurements. The resulting HD IVUS images were segmented with 0.5 mm intervals using echoPlaque 4.0 software (INDEC Medical Systems) and imported into Rhinoceros for the measurement of mean lumen diameter (MLD) and lumen area. To evaluate the IVUS scan reproducibility, each model was scanned twice by the same clinical operator. Additionally, the images were segmented by an independent investigator who was blinded to the initial imaging to assess intraoperator variability.

#### OCT image acquisition and analysis

The silicone models were then imaged using OCT with a Dragonfly OPTIS catheter and OPTIS imaging system operating in Ultreon 2.0 software. Technical specifications and device parameters for OCT are summarized in Supplemental Table S1. OCT pullback was performed at a speed of 36 mm/s, with simultaneous injection of a contrast agent. For OCT, coregistration was performed by identifying the same landmarks on OCT images, ensuring reliable and accurate coregistration. The OCT images were segmented with 0.5 mm intervals using echoPlaque software and imported into Rhinoceros for the measurement of MLD and lumen area. To evaluate OCT scan reproducibility, each model was scanned twice by the same clinical operator. Additionally, the OCT images were segmented by an independent investigator who was blinded to the initial imaging, to assess intraoperator variability.

### Clinical investigation

Sequential HD IVUS and OCT imaging studies were performed in 12 coronary arteries obtained from 9 male patients, who were referred to the catheter laboratory for stable ischemic heart disease. The mean age ± SD of the patients was 56.4 ± 5 years. The imaged arteries were as follows: left anterior descending artery (n = 4), left circumflex artery (n = 3), and right coronary artery (n = 5).

#### HD IVUS image acquisition and analysis

High-definition intravascular ultrasound imaging was done using a 60 MHz OPTICROSS HD 6 catheter with AVVIGO+ system for 3 coronary arteries, and a 60 MHz OPTICROSS HD 6 catheter with POLARIS system for 9 coronary arteries. The imaging catheter was advanced through a 6F guiding catheter, with pullback performed at a constant speed of 1.0 mm/s. Each pullback run was analyzed offline, and the lumen was segmented with 0.5 mm intervals using Echoplaque software. Side branches served as anatomical landmarks to ensure consistent analysis of the same vessel regions and to coregister the HD IVUS pullbacks with those obtained from OCT. For each segmented HD IVUS frame, the MLD and lumen area were calculated by Rhinoceros.

#### OCT image acquisition and analysis

Optical coherence tomography imaging was performed on the same coronary segments using the Dragonfly OPTIS catheter and OPTIS imaging system operating in Ultreon 2.0 software for 3 coronary arteries and with Dragonfly OPTIS catheter and AptiVue software for 9 coronary arteries. A 6F guiding catheter was used to advance the catheter, and OCT pullbacks were conducted at a speed of 36 mm/s with contrast injection. Each pullback run was analyzed offline, and the lumen was segmented with 0.5 mm intervals using echoPlaque software. For each segmented OCT frame, the MLD and lumen area were calculated by Rhinoceros.

### Comparison metrics

For the silicone models, HD IVUS and OCT were compared with μCT serving as the ground truth using MLD and lumen area as comparison metrics. These same metrics were also used to compare HD IVUS and OCT in clinical cases. Additionally, a comprehensive qualitative comparison of anatomical and morphological characteristics, including coronary artery structure, plaque composition, and various pathologies was conducted between HD IVUS and OCT images in the clinical data set.

### Statistical analysis

Statistical analyses were performed using GraphPad Prism 8.0 (GraphPad Inc). Bland Altman analysis was used for the comparison between HD IVUS, OCT, and μCT to show the mean difference ± SD between the MLD and lumen area of the different intracoronary imaging modalities. The reproducibility of HD IVUS and OCT scanning was also assessed using Bland Altman analysis.

## Results

### Silicone model quantitative analysis

**Figure 1:**
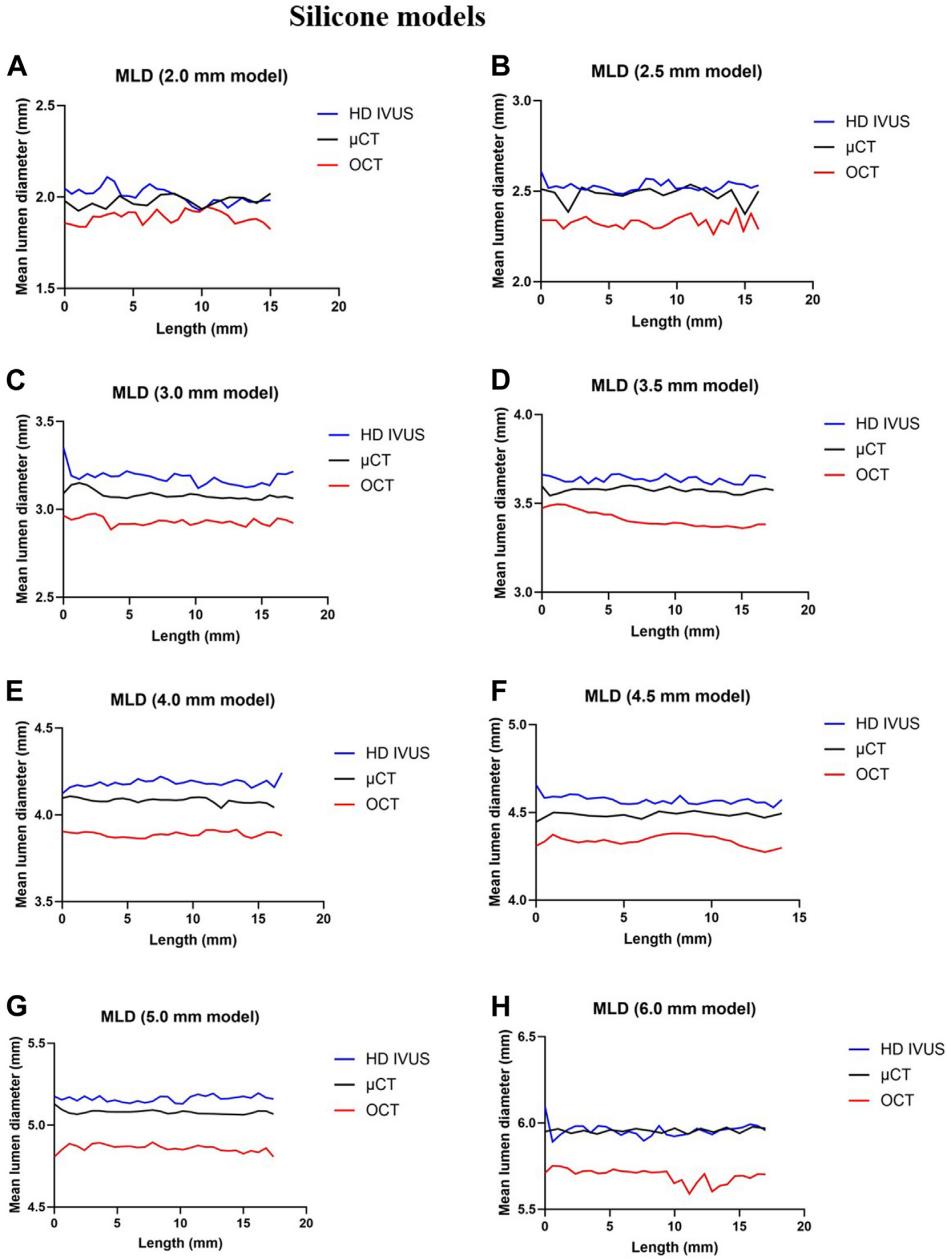
**Lumen diameters by high-definition intravascular ultrasound (HD IVUS), optical coherence tomography (OCT), and microcomputed tomography (μCT) across different silicone models.** (**A**) 2.0 mm, (**B**) 2.5 mm, (**C**) 3.0 mm, (**D**) 3.5 mm, (**E**) 4.0 mm, (**F**) 4.5 mm, (**G**) 5.0 mm, and (**H**) 6.0 mm models. MLD, mean lumen diameter.

**Figure 2:**
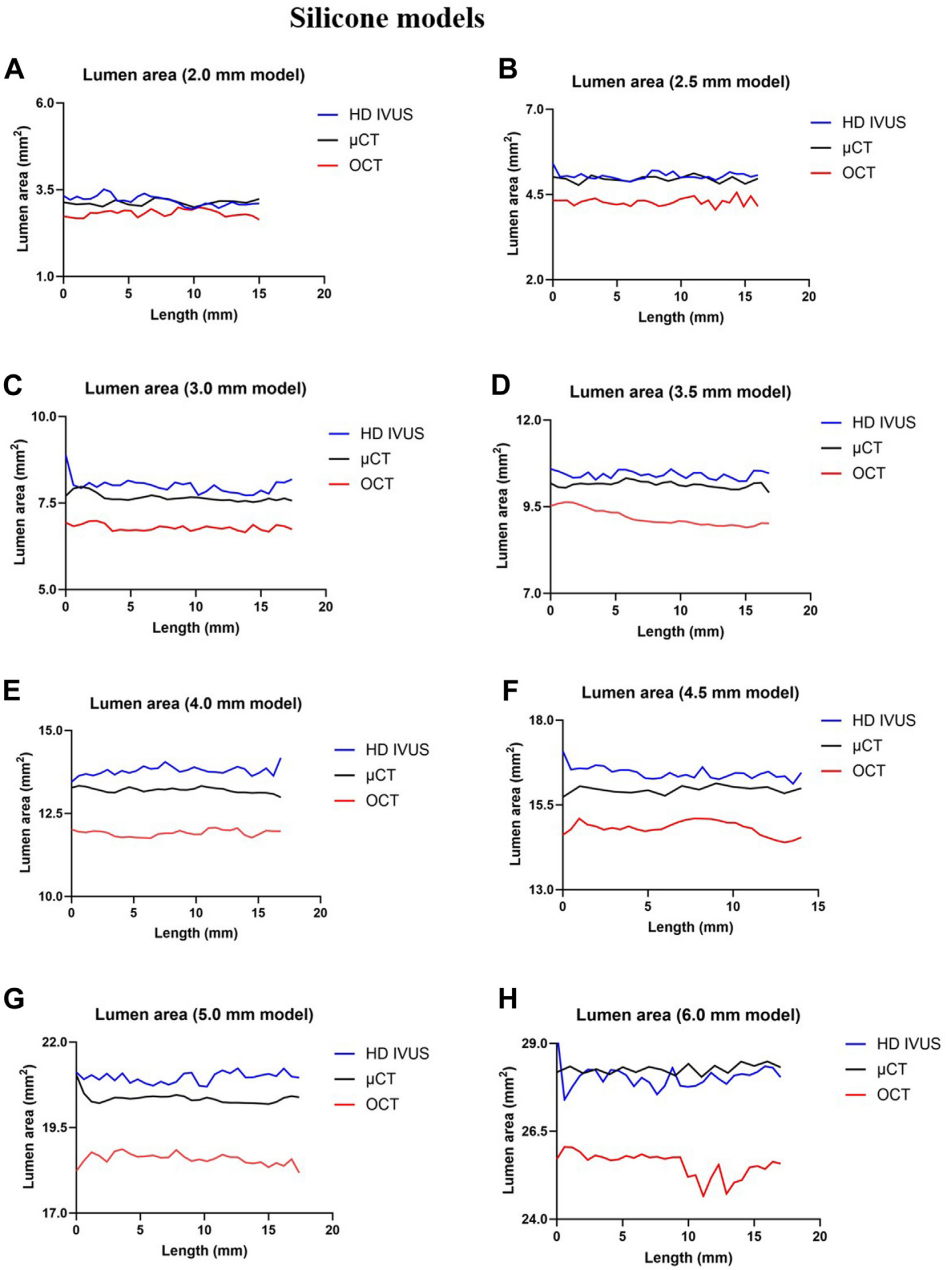
**Lumen areas by high-definition intravascular ultrasound (HD IVUS), optical coherence tomography (OCT), and microcomputed tomography (μCT) across different silicone models.** (**A**) 2.0 mm, (**B**) 2.5 mm, (**C**) 3.0 mm, (**D**) 3.5 mm, (**E**) 4.0 mm, (**F**) 4.5 mm, (**G**) 5.0 mm, and (**H**) 6.0 mm models.

**Figure 3:**
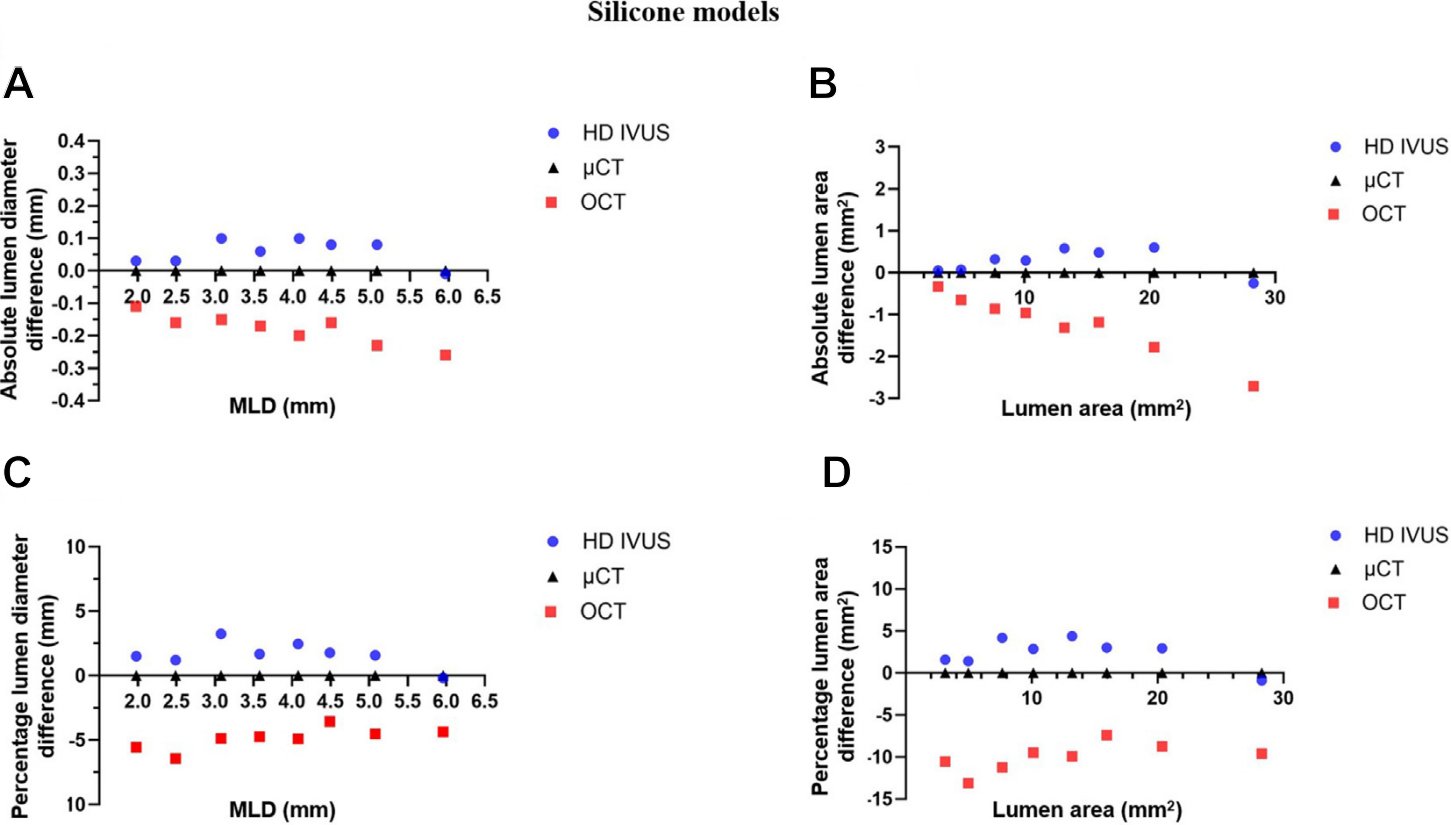
**Absolute and percentage lumen diameter and area differences of high-definition intravascular ultrasound (HD IVUS) and optical coherence tomography (OCT) vs microcomputed tomography (μCT) in silicone models.** (**A**) Absolute difference of HD IVUS and OCT compared to μCT of mean lumen diameter (MLD); (**B**) absolute difference of HD IVUS and OCT compared to μCT of lumen area; (**C**) percentage difference of HD IVUS and OCT compared to μCT of MLD; (**D**) percentage difference of HD IVUS and OCT compared to μCT of lumen area.

**Figure 4:**
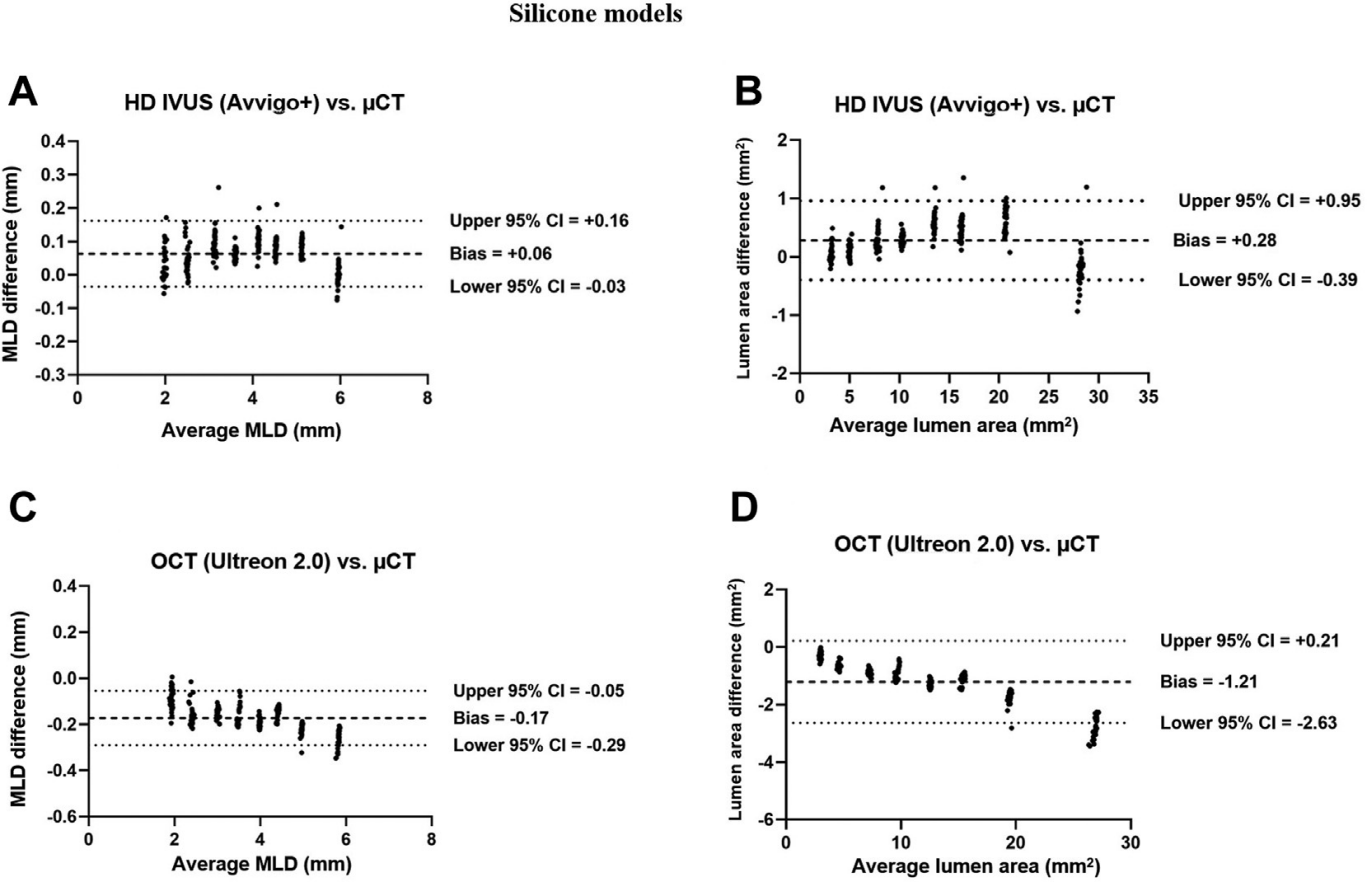
**Bland Altman analysis of high-definition intravascular ultrasound (HD IVUS), optical coherence tomography (OCT) vs microcomputed tomography (μCT) in silicone models.** (**A**) HD IVUS compared with μCT of mean lumen diameter (MLD); (**B**) HD IVUS compared with μCT of lumen area; (**C**) OCT compared with μCT of MLD; (**D**) OCT compared with μCT of lumen area.

Figures 1 and 2 compared MLD and lumen area between HD IVUS (OPTICROSS HD/AVVIGO+) and OCT (Dragonfly OPTIS/ OPTIS/Ultreon 2.0), using μCT as the ground truth for all silicone models (Supplemental Table S2). The absolute and percentage differences in MLD and lumen area between HD IVUS, OCT, and μCT are summarized in Figure 3. Figure 4 presents the Bland Altman analysis of HD IVUS and OCT compared to μCT. HD IVUS consistently overestimated silicone model dimensions by +0.06 ± 0.05 mm (error = +1.7%) for MLD and +0.28 ± 0.34 mm^2^ (error = +2.5%) for lumen area compared to the ground truth of μCT. Conversely, OCT consistently underestimated the lumen dimensions by –0.17 ± 0.06 mm (error = –4.7%) for MLD and –1.2 ± 0.72 mm^2^ (error = –10%) for lumen area. The underestimation of lumen dimensions by OCT was greater for lumen diameters ≥5.0 mm by –0.24 ± 0.03 mm (error = –4.5%) for MLD and –2.24 ± 0.56 mm^2^ (error = –9.5%) for lumen area. Overall, HD IVUS demonstrated a higher level of agreement with μCT compared to OCT. The mean difference between HD IVUS and OCT was +0.23 ± 0.06 mm (+6.5% difference) for MLD and +1.49 ± 0.74 mm^2^ (+13% difference) for the lumen area (Supplemental Figure S1).

The reproducibility of HD IVUS and OCT revealed high agreement between different scans for both imaging modalities. For HD IVUS, the reproducibility bias was –0.006 mm (95% CI, –0.07 to +0.06 mm) for MLD and –0.02 mm^2^ (95% CI, –0.47 to +0.43 mm^2^) for lumen area. For OCT, the reproducibility bias was +0.02 mm (95% CI, –0.09 to +0.13 mm) for MLD and +0.16 mm^2^ (95% CI, –0.76 to +0.08 mm^2^) for lumen area (Supplemental Figure S2).

### Clinical investigations

**Figure 5:**
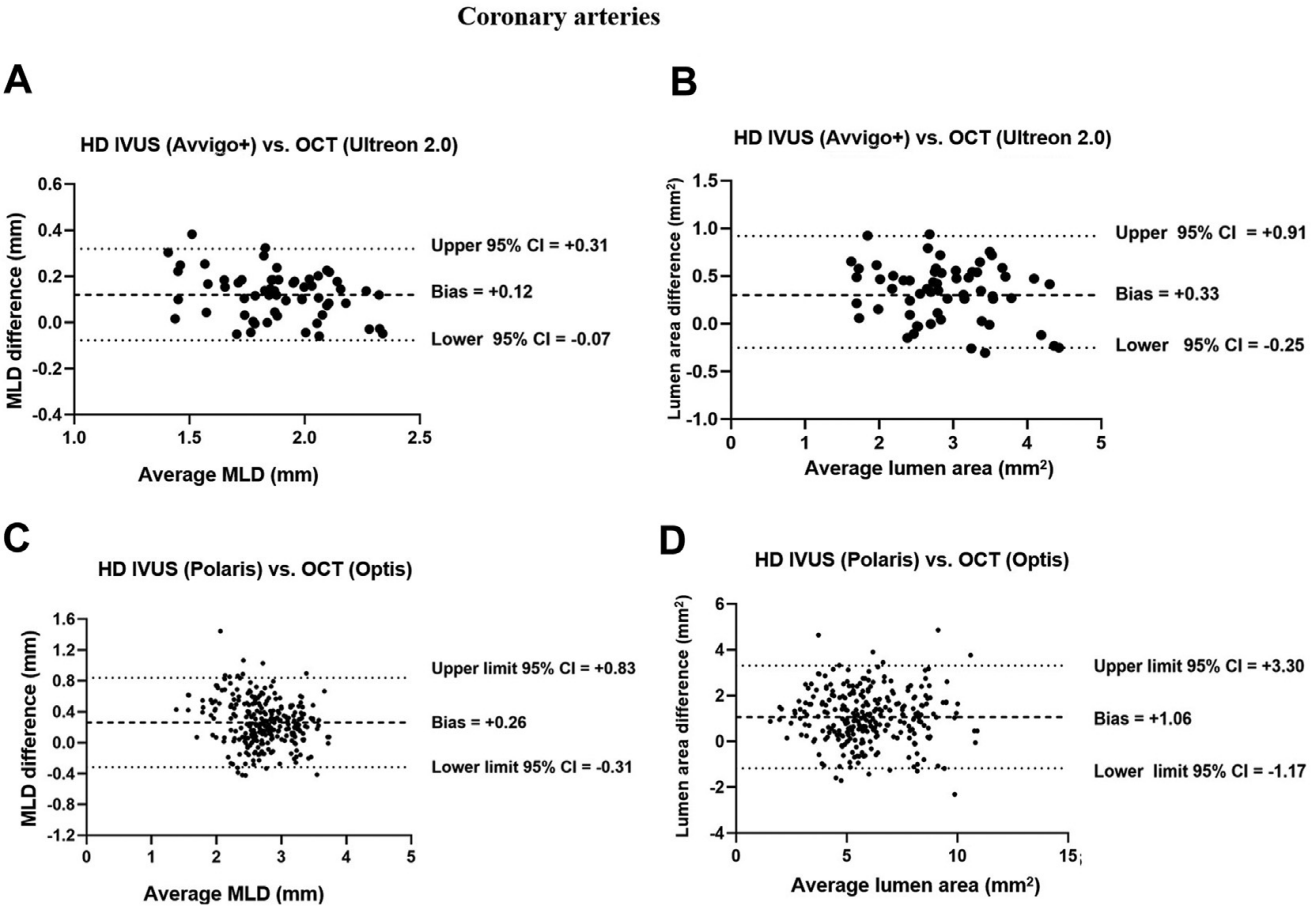
**Bland Altman analysis of high-definition intravascular ultrasound (HD IVUS) compared with optical coherence tomography (OCT) of mean lumen diameter (MLD) and lumen area for coronary arteries.** (**A**) HD IVUS (AVVIGO+) compared with OCT (Ultreon 2.0) of MLD; (**B**) HD IVUS (AVVIGO+) compared with OCT (Ultreon 2.0) of lumen area; (**C**) HD IVUS (POLARIS) compared with OCT (AptiVue) of MLD; (**D**) HD IVUS (POLARIS) compared with OCT (AptiVue) of lumen area.

For the 3 coronary arteries scanned with the latest HD IVUS (OPTICROSS HD/AVVIGO+) and OCT (Dragonfly OPTIS/OPTIS/Ultreon 2.0) systems, the mean difference in MLD was +0.12 mm (95% CI, –0.11 to +0.34) and mean difference in lumen area was +0.33 mm^2^ (95% CI, –0.34 to +0.99) as shown in Figure 5A and B (Supplemental Table S3).

For the 9 coronary arteries scanned with the earlier HD IVUS (OPTICROSS HD/POLARIS) and OCT (Dragonfly OPTIS/AptiVue), HD IVUS yielded larger measurements of MLD with a mean difference of +0.26 mm (95% CI, –0.32 to +0.84) and lumen area with a mean difference of +1.06 mm^2^ (95% CI, –1.18 to +3.30) as shown in Figure 5C and D (Supplemental Table S3).

### Morphological analysis

#### Arterial morphology

Using OCT as a reference, HD IVUS was able to identify fine structures with high precision as described below:

##### Lumen

Optical coherence tomography required blood clearance, which was achieved by contrast injection. Thus, the lumen in OCT appeared clear and black. On HD IVUS, lumen had a speckled appearance (Supplemental Figure S3).

##### Wall

All 3 wall layers were visible in healthy arterial segments on both HD IVUS and OCT (Supplemental Figure S3). In diseased vessel segments, HD IVUS allowed the demarcation of the outer boundary, which was challenging with OCT.

#### Plaque constituents

##### Lipid tissue

On OCT, lipid tissue appeared as a signal-poor region with poorly delineated borders, fast signal drop-off, little or no light backscattering, and high light attenuation. On HD IVUS, lipid-rich tissue or necrotic core within a plaque appeared as homogenously echolucent regions with irregular or poorly defined borders.

##### Fibrous tissue

On OCT, fibrous plaques appeared as relatively homogeneous, signal-rich regions, exhibiting high backscattering of infrared light and low attenuation. On HD IVUS, fibrous tissue appeared moderately echodense, with a heterogeneous texture, without shadows.

##### Calcified tissue

On OCT, calcified plaques appeared as signal-poor, with low backscatter, high penetration, heterogeneous structures with clearly demarcated tissue borders, and the absence of acoustic shadows. On HD IVUS, calcium appeared as highly echogenic tissue. However, the acoustic shadowing from calcified tissue hampered visualization behind calcium; hence, calcium thickness could not be assessed.

#### Plaque types and other fine structures

##### Thin cap fibroatheroma

**Figure 6:**
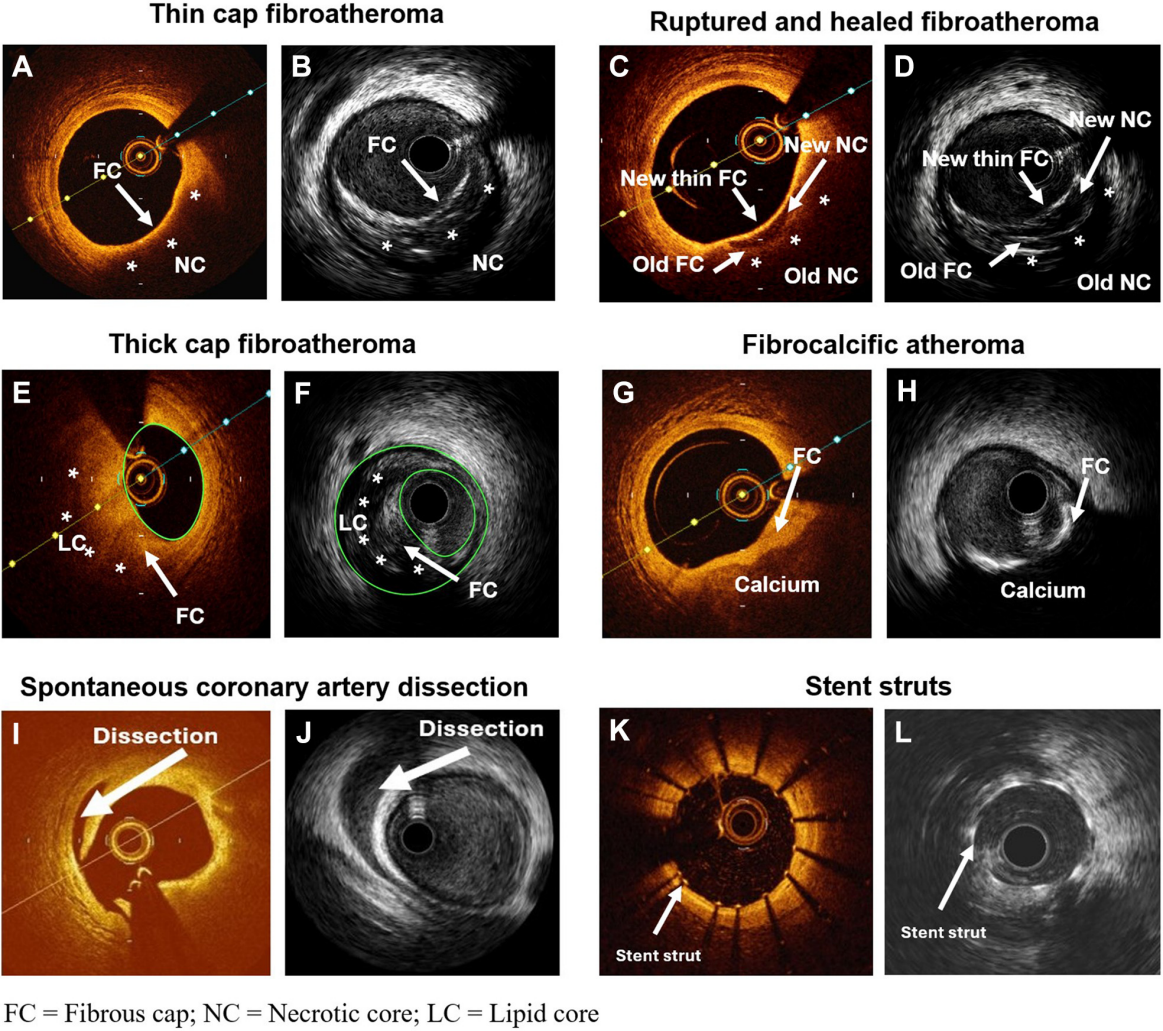
**Fine wall and plaque structures on intravascular ultrasound (IVUS) vs optical coherence tomography (OCT).** (**A**) Thin-cap fibroatheroma on OCT; (**B**) thin cap fibroatheroma on IVUS; (**C**) ruptured and healed fibroatheroma on OCT; (**D**) ruptured and healed fibroatheroma on IVUS; (**E**) thick cap fibroatheroma on OCT; (**F**) thick cap fibroatheroma on IVUS; (**G**) fibrocalcific atheroma on OCT; (**H**) fibrocalcific atheroma on IVUS; (**I**) spontaneous coronary artery dissection on OCT; (**J**) spontaneous coronary artery dissection on IVUS; (**K**) stent strut on OCT; (**L**) stent strut on IVUS. FC, fibrous cap; LC, lipid core; NC, necrotic core.

In OCT, the overlying fibrous cap appeared as a signal-rich thin layer overlying the signal-poor lipid-rich core. Similar to OCT, HD IVUS visualized the thin fibrous cap (echodense structure) overlying the echolucent lipid-rich pool (Figure 6A and B).

##### Ruptured and healed fibroatheroma

In OCT, ruptured fibroatheroma appeared as a discontinuity of fibrous cap, covered by a new fibroatheroma. HD IVUS similarly visualized this plaque structure (Figure 6C and D).

##### Thick-cap fibroatheroma

Both OCT and HD IVUS precisely visualized the thick cap fibroatheroma (Figure 6E and F).

##### Fibrocalcific plaque

On OCT, fibrocalcific plaque appeared as signal-poor with sharp borders and low attenuation covering a signal-poor lipid core. On HD IVUS, fibrocalcific plaque was visualized as a highly echogenic structure with significant acoustic shadowing that obscured imaging of deeper tissue layers (Figure 6G and H).

##### Spontaneous coronary artery dissection

The dissection was visible on both OCT and HD IVUS. Intimal tear and dissection flaps were better detected on OCT, whereas HD IVUS visualized the full extent of the intramural hematoma and delineated the true and false lumen (Figure 6I and J).

##### Stent struts

Stent struts were more clearly visible with OCT. In HD IVUS, stent struts appeared equally echogenic to calcium, with slight acoustic shadowing (Figure 6K and L).

## Discussion

This study provided a comprehensive comparative analysis between the following: (1) The latest HD IVUS and OCT technologies in silicone models against μCT, which was used as ground truth; and (2) HD IVUS against OCT in clinical cases (Central Illustration). The main findings of the study are:

1. In silicone models, HD IVUS consistently overestimated lumen dimensions by +0.06 ± 0.05 mm (error = +1.7%) for MLD and +0.28 ± 0.34 mm^2^ (error = +2.5%) for the lumen area, compared to the ground truth of μCT.
2. Compared to μCT, OCT consistently underestimated the lumen dimensions by –0.17 ± 0.06 mm (error = –4.7%) for MLD and –1.2 ± 0.72 mm^2^ (error = –10%) for lumen area. This underestimation was more pronounced for lumen diameters ≥5.0 mm (–0.24 ± 0.03 mm [error = –4.5%] for MLD and –2.24 ± 0.56 mm^2^ [error = –9.5%] for lumen area).
3. Overall, in silicone models, HD IVUS demonstrated greater accuracy in measuring lumen dimensions compared to OCT.
4. In clinical cases, the earlier HD IVUS (OPTICROSS HD/POLARIS) overestimated lumen size compared to OCT (Dragonfly OPTIS/AptiVue) by +0.26 ± 0.29 mm for MLD and +1.06 ± 1.14 mm^2^ for lumen area, whereas the latest HD IVUS (Opticross HD/AVVIGO+) overestimated lumen size compared to OCT (Dragonfly OPTIS/OPTIS/Ultreon 2.0) by only +0.12 ± 0.11 mm for MLD and +0.33 ± 0.34 mm^2^ for lumen area.
5. Similar to OCT, HD IVUS accurately identified fine structures and pathologies of the arterial wall, including thin fibrous cap, dissection, and stent struts, suggesting its enhanced imaging resolution.

**Central illustration:**
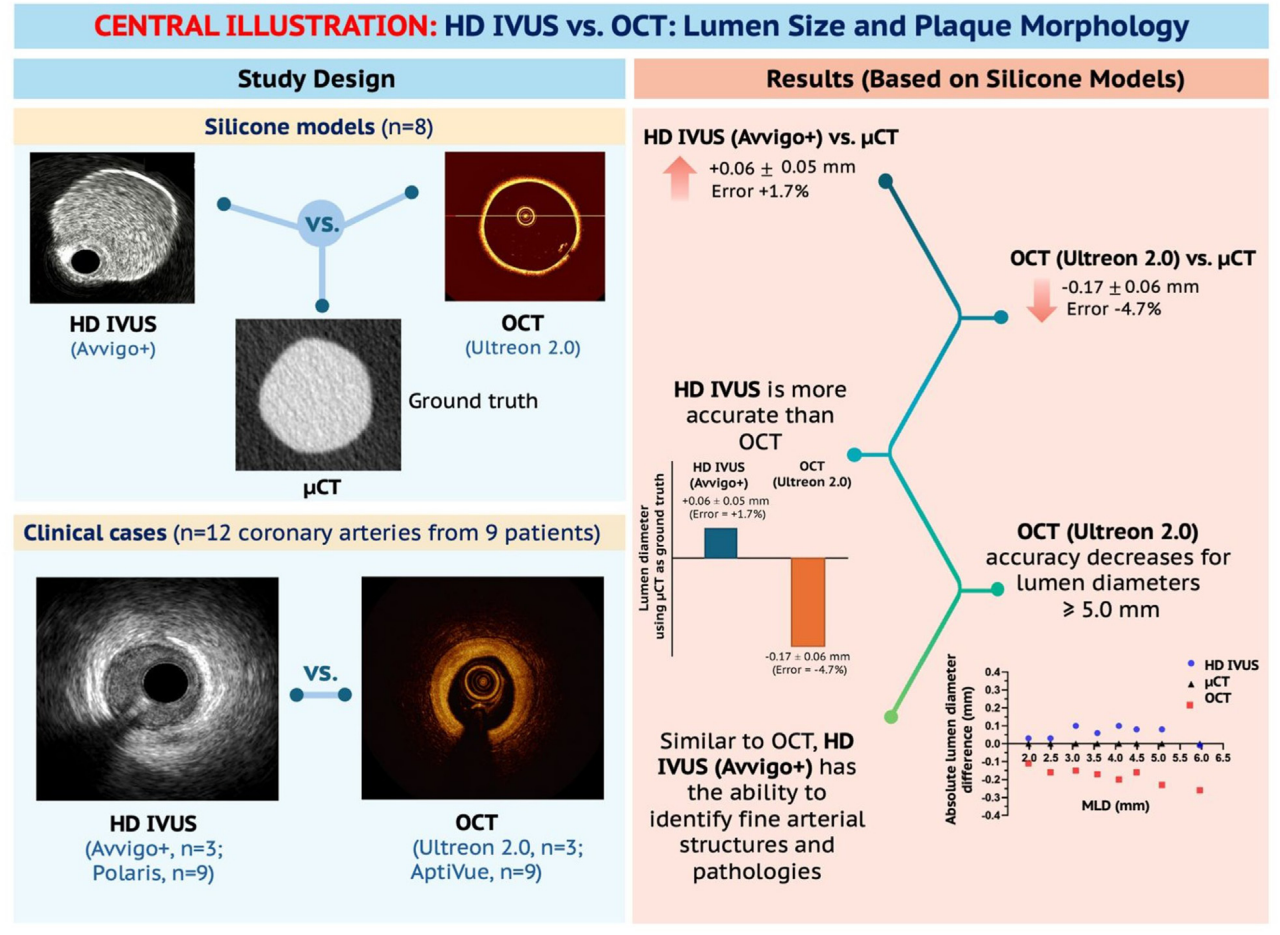
**Comparison of lumen size and plaque morphology between high-definition intravascular ultrasound (HD IVUS) and optical coherence tomography (OCT).** μCT, microcomputed tomography; MLD, mean lumen diameter.

HD IVUS (60 MHz) demonstrates improved accuracy over traditional IVUS (20-40 MHz) due to its higher spatial resolution.^16^ Although many studies have compared IVUS and OCT using traditional IVUS (20/40/45 MHz), only 3 studies (2 in vitro and 1 clinical) have compared HD IVUS with OCT^6^^,^^9^^,^^10^ (Supplemental Table S4). All 3 of them used earlier HD IVUS (OPTICROSS HD/iLab; Kodama, ACIST) and OCT (Dragonfly OPTIS/AptiVue) technologies. One of the in vitro studies created cylindrical phantoms with known diameters ranging from 1.5 to 5.0 mm and noted an overestimation of lumen diameter by +0.12 ± 0.01 mm by HD IVUS (OPTICROSS HD/iLab), and underestimation of lumen diameter by –0.012 ± 0.01 mm by OCT (Dragonfly OPTIS/AptiVue).^9^ It is questionable whether the known dimensions of the phantoms represented the actual dimensions after manufacturing when compared to the computer-aided design dimensions. The other in vitro study used a 3D printed patient-specific left main bifurcation and reported a mean difference of +0.44 ± 0.25 mm in lumen diameter by HD IVUS (OPTICROSS HD/iLab) compared with OCT (Dragonfly OPTIS/AptiVue).^10^ That study did not include objective ground truth lumen measurements. The clinical study compared HD IVUS (Kodama) and OCT (Dragonfly OPTIS/AptiVue) in 29 coronary arteries from 29 patients and revealed a rather small mean difference of −0.049 ± 0.35 mm^2^ in lumen area,^6^ contrasting the findings from previous in vitro studies. Our study advances the current state of the art with the following notable innovations: (1) we used the latest HD IVUS (OPTICROSS HD/AVVIGO+) and OCT (Dragonfly OPTIS/OPTIS/Ultreon 2.0) technologies; (2) we conducted a comprehensive analysis that included both experimental (silicone models) and clinical cases; (3) we used μCT as ground truth which none of the previous studies used; and (4) we compared the in vitro silicone models across a wide range of diameters (2.0-6.0 mm). The improved performance of HD IVUS (OPTICROSS HD/AVVIGO+) over OCT shown in our study might be attributed to the advanced postimaging processing capabilities of the latest AVVIGO+ system.

Interestingly, the differences observed between the latest imaging systems (HD IVUS AVVIGO+ vs OCT Ultreon 2.0: +0.12 mm for MLD, and +0.33 mm^2^ for lumen area) were significantly smaller—approximately half—compared with those observed with older-generation systems (HD IVUS POLARIS vs OCT AptiVue: +0.26 mm for MLD, and +1.06 mm^2^ for lumen area). This finding suggests that the absolute minimal stent area cutoffs currently employed for IVUS (5.5 mm^2^) may merit reconsideration, potentially lowering the threshold to 5.0 mm^2^ or less.^17^ This perspective, derived from the present analysis, warrants further investigation.

Another significant finding in our study is the ability of HD IVUS (AVVIGO+) to visualize fine arterial wall structures and pathologies. OCT is well-known for its superior imaging resolution, providing clearer and crisper images of arterial wall pathologies. Our study demonstrates that HD IVUS approaches the resolution of OCT while simultaneously maintaining its accuracy with larger lumen diameters. In contrast to this, OCT accuracy diminishes for lumen diameters ≥5.0 mm (Figure 3), a limitation commonly recognized for OCT imaging. Table 1 presents a comparison of HD IVUS vs OCT in characterizing fine structures and pathologies within the arterial wall.

**Table 1:** Imaging of arterial wall pathologies by HD IVUS vs OCT.

Plaque characteristic	HD IVUS	OCT
Lipid	++	++
Necrotic core	+	++
Fibrous cap	+	++
Calcification		
Circumference of calcification	++	++
Depth of calcification	-	++
Thin-cap fibroatheroma	+	++
Thrombus	+	++
Macrophages	-	+
Cholesterol crystals	-	+
Left main coronary artery lesion	++	+
Aorto-ostial lesion	++	-
Coronary dissection	+	++
Stent apposition/expansion	+	++
Stent edge dissection	+	++
Tissue protrusion through the strut	+	++
Neointimal hyperplasia	+	++

-, poor assessment; +, good assessment; ++, excellent assessment; HD IVUS, high-definition intravascular ultrasound; OCT, optical coherence tomography.

The limitations of our study are outlined as follows: (1) the limited number of clinical cases, particularly in the comparison between the AVVIGO+ and Ultreon 2.0 systems; (2) in the qualitative analysis, the operators reviewing the HD IVUS and OCT images were not blinded; however, the use of identical vessel regions for both imaging modalities significantly mitigates the potential for observer bias; (3) in the clinical investigations, a definitive ground truth was unavailable. Consequently, the present study cannot provide conclusive data on the comparative accuracy of HD IVUS and OCT in a clinical context. However, extrapolating our experimental findings—where a well-defined ground truth was utilized—suggests that HD IVUS and OCT exhibit comparable accuracy, with a potential trend indicating that HD IVUS can be more accurate than OCT, especially in large coronary arteries. Nonetheless, further clinical studies are essential to validate these findings. Additionally, the findings of this study are specific to the imaging systems we studied. Although these systems are the most widely used, the results cannot be translated to other imaging systems available in the market.

## Conclusion

Our study demonstrated that the latest high-definition IVUS (OPTICROSS HD/AVVIGO+) tends to overestimate lumen size, whereas OCT (Dragonfly OPTIS/Ultreon 2.0) tends to underestimate it. Overall, our experimental data indicate that HD IVUS provides a more accurate assessment of lumen dimensions compared to OCT. However, in the absence of an absolute clinical ground truth, this study cannot definitively determine the comparative accuracy of HD IVUS and OCT in clinical settings. Extrapolating our experimental findings—based on a well-defined ground truth—suggests that HD IVUS and OCT demonstrate comparable accuracy, with a potential trend favoring HD IVUS, particularly in larger coronary arteries. Further clinical studies are necessary to validate these observations.

Importantly, the differences between the latest HD IVUS and OCT technologies appear to be less pronounced than those observed with earlier-generation systems. This convergence in performance highlights the need to revisit absolute minimal stent area cutoffs defined by IVUS and OCT. Further clinical investigations are warranted to validate these cutoffs and refine guidelines for intracoronary imaging in interventional cardiology.
