# Corrigendum to ‘High-Definition Intravascular Ultrasound Versus Optical Coherence Tomography: Lumen Size and Plaque Morphology’ [Journal of the Society for Cardiovascular Angiography & Interventions; 4 (2025) 102520]

**DOI:** 10.1016/j.jscai.2025.103945

**Published:** 2025-08-22

**Authors:** Wei Wu, Shijia Zhao, Akshat Banga, Yash Vardhan Trivedi, Vineeth S. Dasari, Parth Munjal, Rakshita Ramesh Bhat, Ruben K.A. Tapia-Orihuela, Usama M. Oguz, Hammad Zafar, Haritha Darapaneni, Nikolaos Spilias, Jessica Wagner, Stephen Morin, Amanda DeVos, Paul A. Iaizzo, Akiko Maehara, Evan S. Shlofmitz, Ziad A. Ali, Emmanouil Brilakis, George D. Dangas, Thomas Johnson, Yiannis S. Chatzizisis

**Affiliations:** a Center for Digital Cardiovascular Innovations, Cardiovascular Division, Miller School of Medicine, Miami, Florida; b Department of Chemistry, University of Nebraska–Lincoln, Hamilton Hall, Lincoln, Nebraska; c Visible Heart Laboratories, Department of Surgery and the Institute for Engineering in Medicine, University of Minnesota, Minneapolis, Minnesota; d Division of Cardiovascular Medicine, Columbia University Irving Medical Center, New York, New York; e Division of Cardiovascular Medicine, St. Francis Hospital & Heart Center, Roslyn, New York; f Center for Complex Coronary Interventions, Minneapolis Heart Institute, Minneapolis, Minnesota; g Division of Cardiovascular Medicine, Icahn School of Medicine at Mount Sinai, New York, New York; h Bristol Heart Institute, Translational Health Sciences, University of Bristol, Bristol, United Kingdom

The authors regret that an incorrect version of Figure 6 was submitted for publication. The correct version is shown below. The authors would like to apologize for any inconvenience caused.

**Figure 6:**
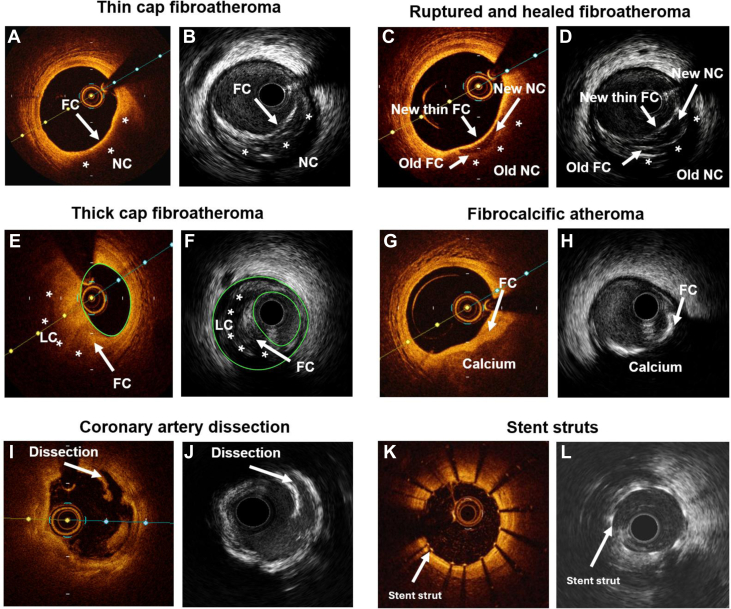
**Fine wall and plaque structures on intravascular ultrasound (IVUS) vs optical coherence tomography (OCT).** (**A**) Thin-cap fibroatheroma on OCT; (**B**) thin-cap fibroatheroma on IVUS; (**C**) ruptured and healed fibroatheroma on OCT; (**D**) ruptured and healed fibroatheroma on IVUS; (**E**) thick-cap fibroatheroma on OCT; (**F**) thick-cap fibroatheroma on IVUS; (**G**) fibrocalcific atheroma on OCT; (**H**) fibrocalcific atheroma on IVUS; (**I**) coronary artery dissection on OCT; (**J**) coronary artery dissection on IVUS; (**K**) stent strut on OCT; (**L**) stent strut on IVUS. FC, fibrous cap; LC, lipid core; NC, necrotic core.

