# Supplementary material for: High-Definition Intravascular Ultrasound Versus Optical Coherence Tomography: Lumen Size and Plaque Morphology
Source: J Soc Cardiovasc Angiogr Interv. 2025 May 1;4(5):102520. doi: 10.1016/j.jscai.2024.102520 (PMC12126068; doi:10.1016/j.jscai.2024.102520)
Supplement: Supplemental Figures [file mmc1.pptx]

## Slide 1
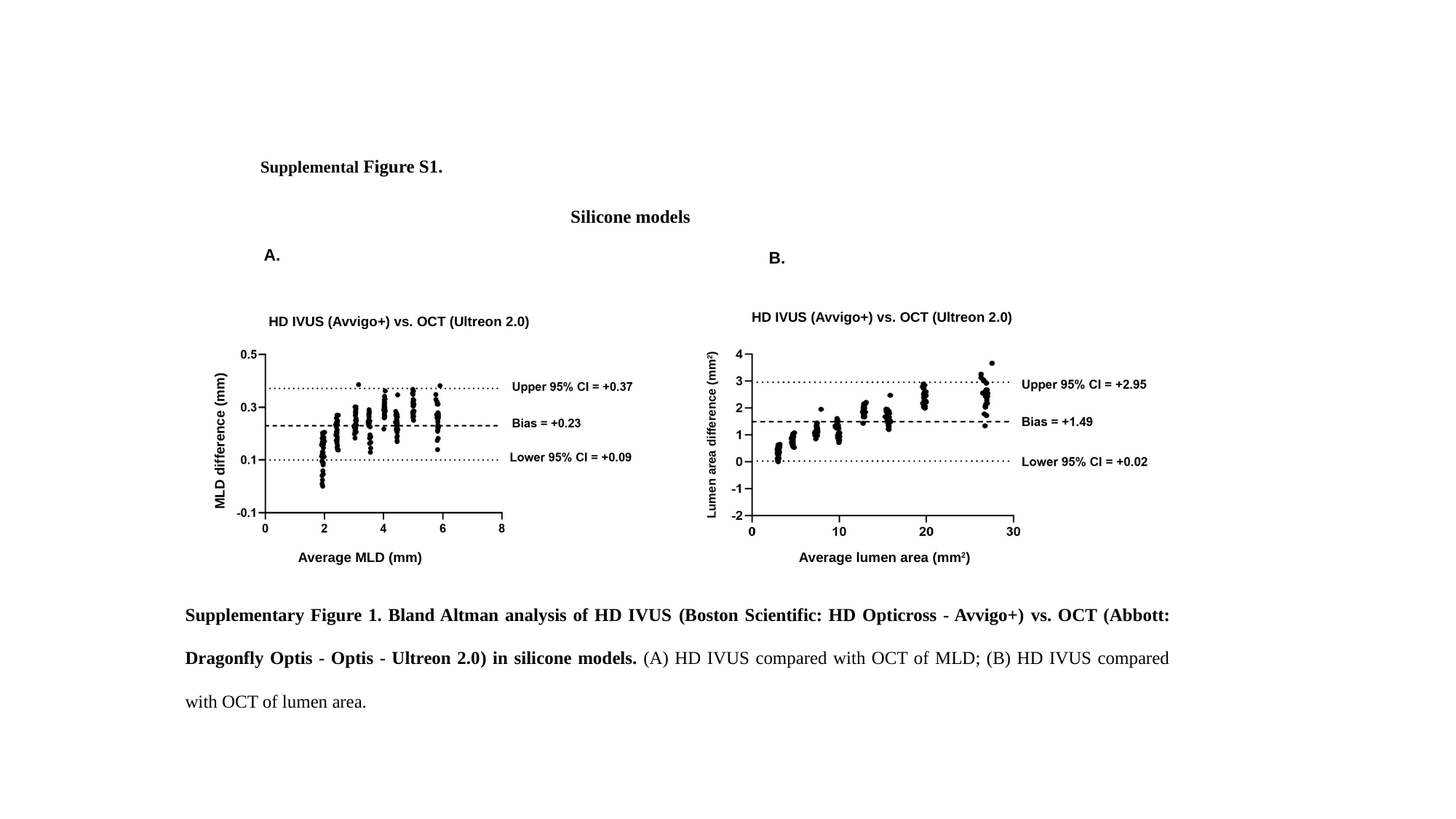

Supplemental Figure S1.
 Silicone models
A.
B.
 Average MLD (mm)
 Average lumen area (mm2)
HD IVUS (Avvigo+) vs. OCT (Ultreon 2.0)
HD IVUS (Avvigo+) vs. OCT (Ultreon 2.0)
MLD difference (mm)
 Lumen area difference (mm2)
Supplementary Figure 1. Bland Altman analysis of HD IVUS (Boston Scientific: HD Opticross - Avvigo+) vs. OCT (Abbott: Dragonfly Optis - Optis - Ultreon 2.0) in silicone models. (A) HD IVUS compared with OCT of MLD; (B) HD IVUS compared with OCT of lumen area.

## Slide 2
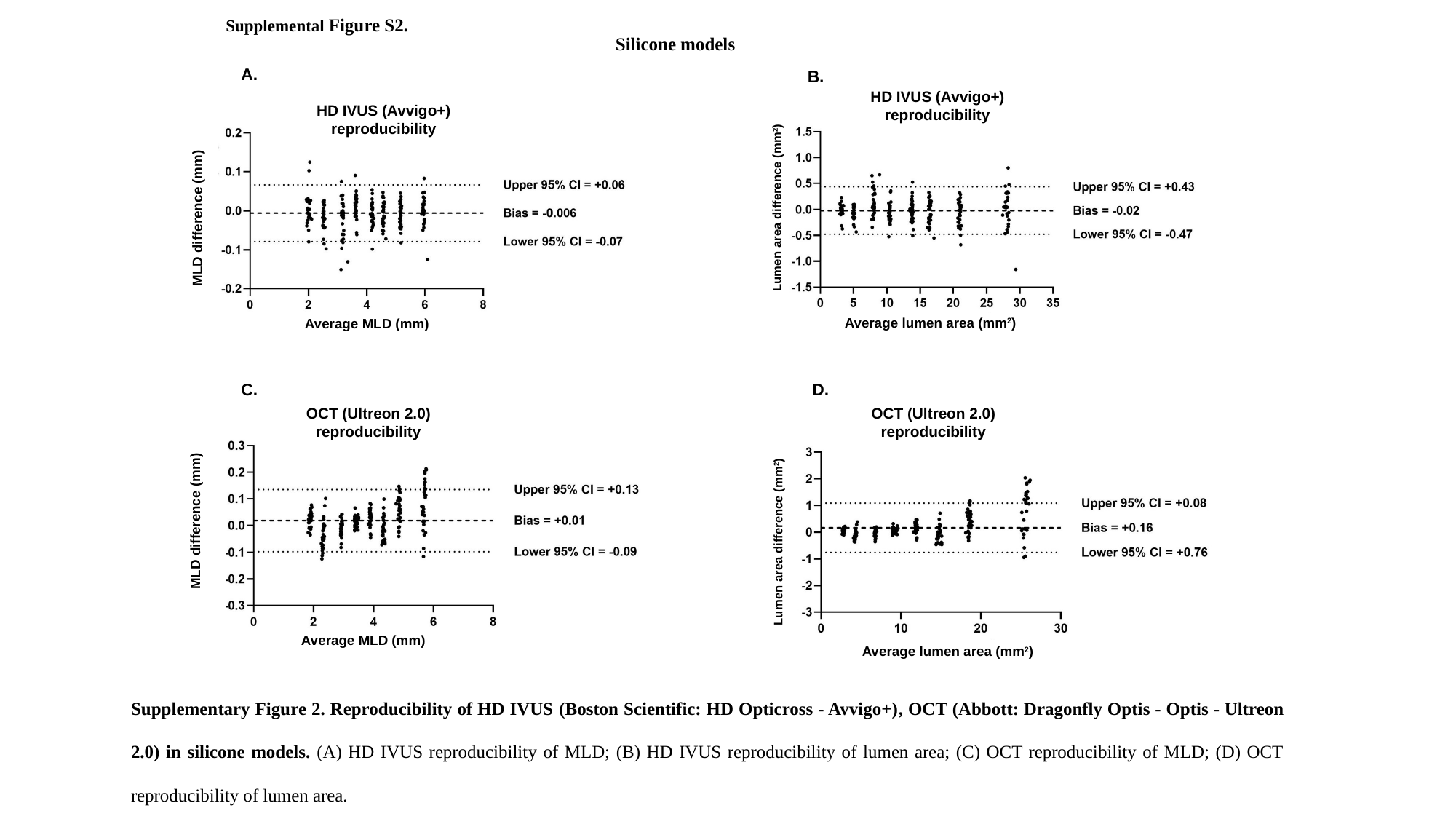

Silicone models
A.
B.
 Average lumen area (mm2)
 Average MLD (mm)
C.
D.
 Average MLD (mm)
 Average lumen area (mm2)
 Supplemental Figure S2.
HD IVUS (Avvigo+) reproducibility
HD IVUS (Avvigo+) reproducibility
MLD difference (mm)
 Lumen area difference (mm2)
OCT (Ultreon 2.0) reproducibility
OCT (Ultreon 2.0) reproducibility
MLD difference (mm)
 Lumen area difference (mm2)
Supplementary Figure 2. Reproducibility of HD IVUS (Boston Scientific: HD Opticross - Avvigo+), OCT (Abbott: Dragonfly Optis - Optis - Ultreon 2.0) in silicone models. (A) HD IVUS reproducibility of MLD; (B) HD IVUS reproducibility of lumen area; (C) OCT reproducibility of MLD; (D) OCT reproducibility of lumen area.

## Slide 3
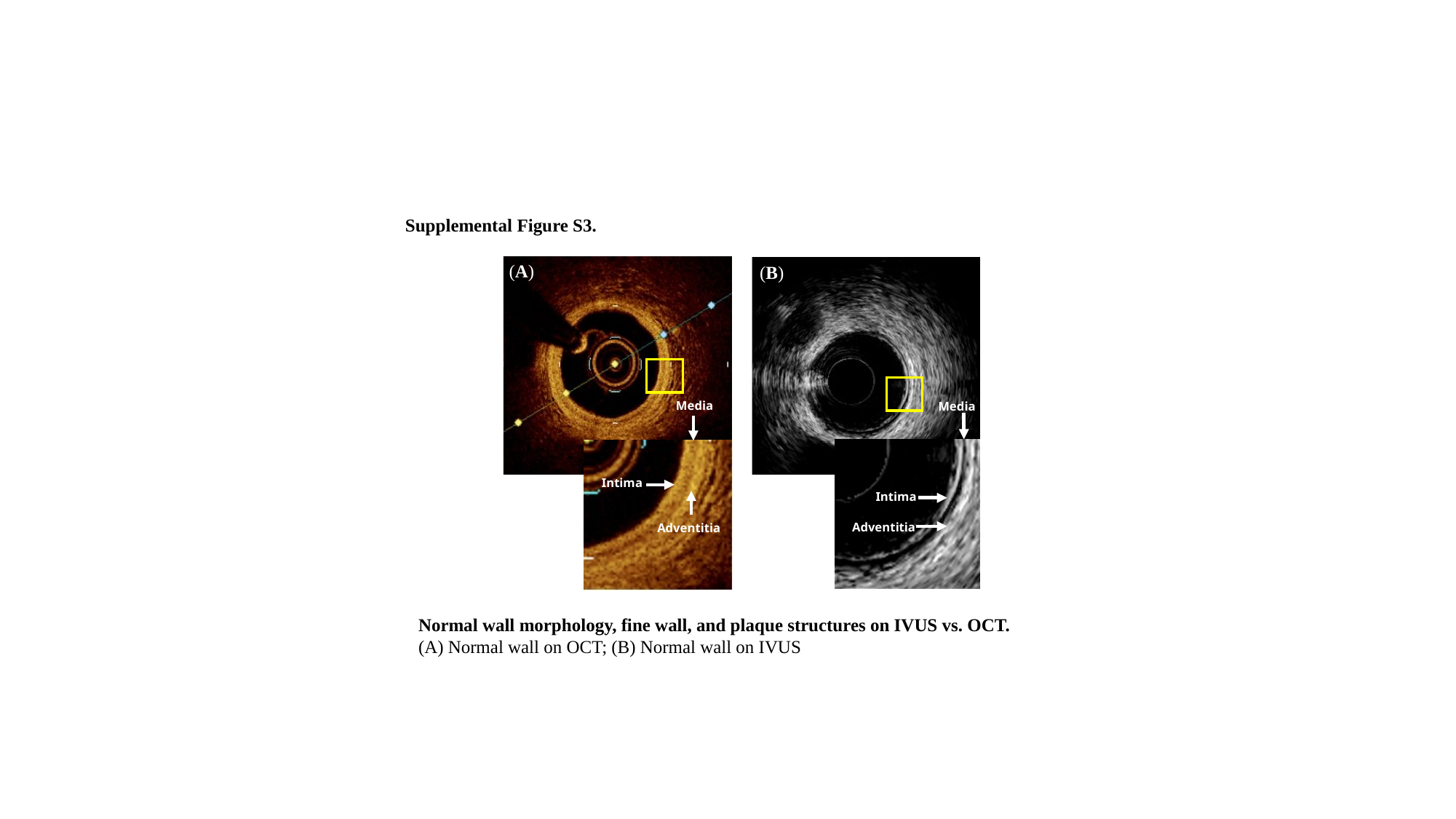

Supplemental Figure S3.
(A)
(B)
B
A
Media
Intima
Adventitia
Adventitia
Media
Media
Intima
Intima
Adventitia
Adventitia
Media
Media
Intima
Intima
Adventitia
Adventitia
Normal wall morphology, fine wall, and plaque structures on IVUS vs. OCT. (A) Normal wall on OCT; (B) Normal wall on IVUS
