# Supplementary material for: High-Definition Intravascular Ultrasound Versus Optical Coherence Tomography: Lumen Size and Plaque Morphology
Source: J Soc Cardiovasc Angiogr Interv. 2025 May 1;4(5):102520. doi: 10.1016/j.jscai.2024.102520 (PMC12126068; doi:10.1016/j.jscai.2024.102520)
Supplement: Supplemental Tables [file mmc2.docx]

**Supplementary Tables**

# **Supplemental Table S1. Comparison of technical and device parameters between HD IVUS and OCT**

| **Characteristic** | **HD IVUS (Boston Scientific)** | **OCT (Abbott)** |
| --- | --- | --- |
| Type of wave | Ultrasound | Near-infrared light |
| Frequency or Wavelength | 60 MHz | 1.25-1.35μm |
| Axial Resolution (μm) | 22 | 10-20 |
| Lateral Resolution (μm) | >50 | <50 |
| Tissue Penetration depth (mm) | 6 | 1-3 |
| Distance between adjacent  frames (mm) | 0.017-0.267 | 0.10-0.25 |
| Frame rate (fps) | 30 | 100 |
| Pullback speed (mm/s) | 0.5-8.0 | 20-40 |
| Maximum pullback length (mm) | 100-150 | 75-150 |

HD IVUS: High-definition intravascular ultrasound; OCT: Optical coherence tomography; EEM: External elastic membrane

# **Supplemental Table S2. Comparison of HD IVUS, OCT and µCT in silicone models**

| **Model size** | **MLD (mm)** | | | **Area (mm2)** | | |
| --- | --- | --- | --- | --- | --- | --- |
|  | HD IVUS | OCT | µCT | HD IVUS | OCT | µCT |
| 2.0 mm | 2.01±0.04 | 1.87±0.03 | 1.98±0.03 | 3.18±0.13 | 2.80±0.09 | 3.13±0.08 |
| 2.5 mm | 2.52±0.02 | 2.33±0.03 | 2.49±0.04 | 5.03±0.09 | 4.31±0.12 | 4.96±0.09 |
| 3.0 mm | 3.18±0.03 | 2.93±0.03 | 3.08±0.02 | 7.98±0.15 | 6.80±0.13 | 7.66±0.11 |
| 3.5 mm | 3.64±0.02 | 3.41±0.03 | 3.58±0.01 | 10.42±0.11 | 9.17±0.17 | 9.17±0.17 |
| 4.0 mm | 4.18±0.02 | 3.88±0.04 | 4.08±0.02 | 13.78±0.12 | 11.89±0.26 | 13.20±0.08 |
| 4.5 mm | 4.57±0.02 | 4.33±0.07 | 4.49±0.02 | 16.43±0.15 | 14.77±0.49 | 14.77±0.49 |
| 5.0 mm | 5.16±0.02 | 4.85±0.04 | 5.08±0.01 | 20.95±0.15 | 18.57±0.33 | 18.57±0.33 |
| 6.0 mm | 5.95±0.02 | 5.70±0.05 | 5.96±0.01 | 28.02±0.25 | 25.56±0.29 | 28.27±0.13 |

HD IVUS: High-definition intravascular ultrasound; OCT: Optical coherence tomography; µCT: Micro-

computed tomography; MLD: Mean lumen diameter

**Supplemental Table S3. Comparison of HD IVUS (Boston Scientific: HD Opticross - Avvigo+)** **vs. OCT (Abbott: Dragonfly Optis - Optis - Ultreon 2.0) for n=3 coronary arteries and HD IVUS (Boston Scientific: HD Opticross - Polaris) vs. OCT (Abbott: Dragonfly Optis - AptiVue) for n=9 coronary arteries.**

|  | **MLD (mm)** | **Lumen Area (mm^2^)** |
| --- | --- | --- |
| HD IVUS (Avvigo+) | 1.94±0.21 | 3.04±0.66 |
| OCT (Ultreon 2.0) | 1.82±0.25 | 2.71±0.74 |
|  | MLD (mm) | **Lumen Area (mm^2^)** |
| HD IVUS (Polaris) | 2.86±0.44 | 6.53±1.94 |
| OCT (Aptivue) | 2.60±0.50 | 5.47±1.96 |

HD IVUS: High-definition intravascular ultrasound; OCT: Optical coherence tomography; µCT:

Micro-computed tomography; MLD: Mean lumen diameter

**Supplemental Table S4. Review of HD IVUS vs. OCT comparison studies**

| Study | Number of cases | HD IVUS device | OCT device | Lumen diameter | Lumen area | Limitations |
| --- | --- | --- | --- | --- | --- | --- |
| Wu et al  (Current study) | Silicone models (n=8)  Clinical cases (n= 11) | HD IVUS 60 Hz  - Boston Scientific: HD Opticross - Avvigo+  HD Opticross - Polaris | - Abbott: Dragonfly Optis - Optis - Ultreon 2.0  Dragonfly Optis - AptiVue | Silicone models:  HD IVUS (Avvigo+) vs. µCT  +0.06±0.05 mm  OCT (Ultreon 2.0) vs. µCT  -0.17±0.06 mm  Clinical cases:  HD IVUS vs. OCT (Avvigo+ vs. Ultreon 2.0)  +0.12 mm  HD IVUS vs. OCT (Polaris vs. AptiVue)  +0.26 mm | Silicone models:  HD IVUS (Avvigo+) vs. µCT  +0.28±0.34 mm²  OCT (Ultreon 2.0) vs. µCT  -1.2±0.72 mm²  Clinical cases:  HD IVUS vs. OCT (Avvigo+ vs. Ultreon 2.0)  +0.33 mm²  HD IVUS vs. OCT (Polaris vs. AptiVue)  +1.06 mm² | - Limited number of clinical cases  - Non-generalizability of results |
| Garcia- Guimaraes et al (2020) (7) | *In-vivo* (n=29) | HD IVUS 60 MHz  Acist: Kodama | Abbott: Dragonfly Optis - Optis | NA | No significant differences in lumen area estimation | - Small sample size  - Non-generalizability of results |
| Nishi et al (2021) (17) | *In-vitro* (n=5) | HD IVUS 60 MHz  Boston Scientific: HD Opticross - iLab | Abbott: Dragonfly JP - ILUMIEN Optis | HD IVUS vs. Phantom model  +0.12±0.01 mm  OCT vs. Phantom model  -0.012±0.01 mm | NA | - Only *in-vitro* analysis  - It is uncertain whether the known dimensions of the phantoms reflect the actual post-manufacturing dimensions or the computer-aided design dimensions  - Phantom diameters measured by IVUS in saline differ from those measured in blood due to difference in speed of sound between saline and blood |
| Oliveira et al (2023) (18) | *In-vitro* (n=5) | HD IVUS 60 MHz  Boston Scientific: HD Opticross - iLab | Abbott: Dragonfly Optis - Optis | HD IVUS vs. OCT  +0.44±0.25 mm | HD IVUS vs. OCT  +3.01±1.88 mm**^2^** | - No objective ground truth used for comparison between HD IVUS and OCT |

HD IVUS: High-definition intravascular ultrasound; OCT: Optical coherence tomography; MLD: Mean lumen diameter; NA: Not applicable
